# Periconceptional folic acid supplementation and vitamin B_12_ status in a cohort of Chinese early pregnancy women with the risk of adverse pregnancy outcomes

**DOI:** 10.3164/jcbn.16-45

**Published:** 2017-02-08

**Authors:** Ting Yang, Yan Gu, Xiaoping Wei, Xiaohua Liang, Jie Chen, Youxue Liu, Ting Zhang, Tingyu Li

**Affiliations:** 1 Children Nutrition Research Centre, Children’s Hospital of Chongqing Medical University, Ministry of Education Key Laboratory of Child Development and Disorders, China International Science and Technology Cooperation Base of Child Development and Critical Disorders, Chongqing Key Laboratory of Pediatrics, No. 136 Zhongshan Er Rd, Yuzhong District, Chongqing 400014, China; 2 Capital Institute of Pediatrics, No. 2 Yabao Er Rd, Chaoyang District, Beijing 100020, China

**Keywords:** folate, vitamin B_12_, adverse pregnancy outcomes, early pregnancy, newborn

## Abstract

Maternal folate and vitamin B_12_ deficiency predict poor pregnancy outcome. To improve pregnancy outcomes in rural area of China, we investigate rural women’s folic acid supplementation (FAS) status and the associations between maternal vitamin B status during the first trimester and subsequent adverse pregnancy outcomes. We collected the questionnaire information and drew 5 ml of blood from 309 early pregnant rural women. The birth outcomes were retrieved from medical records after delivery. Out of the total, 257 had taken FAS, including 50 before conception (group A) and 207 during the first trimester (group B). The concentration of plasma folate and the RBC folate supplementation groups were obviously higher than that of no-supplementation group (group N, *p*<0.01). The mean vitamin B_12_ levels in FAS group were significantly higher than those in groups N and B (*p*<0.05). Women who delivered SGA or premature infants had reduced plasma folate levels (*p*<0.05) compared with controls. The multiple linear regression models revealed that RBC folate levels affected the infant birth weight (*p*<0.01) and birth length (*p*<0.05). In conclusion, FAS can significantly improve plasma folate and RBC folate levels in childbearing-age women and reduce the risk of subsequent adverse pregnancy outcomes.

## Introduction

Folate and vitamin B_12_ play a vital role in one carbon metabolism which is crucial for the general wellbeing of pregnancy and particularly for fetal growth.^(1)^ It is widely recognised that peri-conceptional FAS reduces the risk of neural tube defects (NTDs) and other congenital malformations. Some observational studies have suggested that reduced circulating folate in pregnancy is associated with increased risk for prematurity, SGA (<10th centile) and other adverse pregnancy outcomes.^(2–5)^

The prevalence of NTDs in China is approximately 2.7 per 1,000 pregnancies, which is significantly higher than that of approximately 1 per 1,000 pregnancies in Caucasian Americans^(6,7)^ Daily consumption of a supplement containing 400 µg folic acid before and during the first trimester of pregnancy has been shown to be effective in preventing both the occurrence of NTDs and numerous adverse pregnancy outcomes.^(6,8,9)^ However, only 3–5% of Chinese childbearing-age women take folic acid, although 20–30% of them know the importance of folic acid in preventing NTDs and some adverse pregnancy outcomes.^(10–12)^ A recent publication stated that further research to measure the effect of folate intake during pregnancy on reducing low birth weight and adverse pregnancy outcomes is an ‘urgent priority’.^(5)^ As there are strategies to reduce the occurrence of these defects by folic acid fortification of the diet are under way in several parts of the world. In 2009, the Chinese provincial government began distributing free peri-conceptional FAS (400 µg per day) to rural women through the Family Planning System and Maternal and Child Health Care System.

Unpublished data from a population-based surveillance system demonstrated that the prevalence of NTDs in China is in rural counties, where women tend to have more children than urban women do;^(12)^ this is of significance because farmers comprise 70% of the total population in China. Tongliang County is a rural district of Chongqing, China and a typical mountainous area with low socioeconomic development. However, most pregnant rural women visit the prenatal clinic not until after the second month of pregnancy; therefore, few receive the supplements when their use is most crucial.

Red blood cell (RBC) and plasma folate concentration levels help in the assessment of folate status in humans. Plasma folate concentrations change immediately after ingestion of folic acid and are therefore commonly measured during the fasting state for bioavailability studies, whereas RBC folate concentrations change slowly, providing a better indication of long-term folate status.^(5,13)^ By contrast, the effects of low vitamin B_12_ status in reproduction are not well defined. Vitamin B_12_ acts as a cofactor for enzyme reactions in one-carbon metabolism and plays a vital role in folate circulation. A study in Bangalore, South India, low maternal vitamin B_12_ concentrations predicted fetal growth retardation.^(14,15)^ One study from China found that inadequate prepregnancy serum vitamin B_12_ status was associated with a 60% increased risk of preterm birth.^(1)^ Folate and vitamin B_12_ status are important determinants of plasma homocysteine and raised total homocysteine levels in maternal blood and amniotic fluid are associated with an increased risk of adverse pregnancy outcomes. This raises the question whether supplementation with folic acid periconceptional supplementation will further improve the pregnancy outcomes and have any potential correlation with Vitamin B_12_ status. Over all, the implications of starting pregnancy and lactation with low vitamin B_12_ status need further investigation.

To our knowledge, no previous study has been conducted in County(i.e., Tongliang County) on self-reported folic acid supplementation and optimal folic acid use (i.e., regularly from 12 weeks before pregnancy through 12 weeks after delivery), especially the concentrations of plasma folate, RBC folate and vitamin B_12_ among women of child-bearing age during the periconceptional period, at the population level. In the present study, our aim was to understand the rates of periconceptional folic acid supplementation and to identify their correlations with blood folate concentrations and adverse pregnancy outcomes of the rural women.

## Materials and Methods

### Study participants

This study was an on-going population-based prospective cohort study. It was conducted in Tongliang Maternal and Children Health Care Hospital of Chongqing, China, which was the only one maternal and children health care hospital in the county. And most of the pregnancy would come to do antenatal examinations. Study participants were local (Tongliang County) women in early pregnancy (gestational age <12 weeks) and 18–35 years old. (Tongliang County, Chongqing, China is a rural district with low socioeconomic development.) Only healthy mothers were admitted into the protocol; the exclusion criteria were pregnancy toxaemia, bronchial asthma, active hepatitis, malignant tumours, chronic renal failure and heart failure. In addition, we had excluded those pregnant women using other dietary supplements (i.e., multivitamins, Fe) during pregnancy(*n* = 4). In the present study, A total of 309 eligible consenting women were recruited and completed the interview, 299 of them contributed a blood sample in early pregnancy. There were 253 mother-infant pairs who remained in the study after delivery, for whom we had complete data on all measurements and variables required for the analysis.

The study protocol was reviewed and approved by the institutional ethical committee of the Capital Institute of Paediatrics, Beijing, China. Informed consent was obtained from all participants before their entry into the study.

### Questionnaire interview

Information on maternal age, educational level, income, demographic information, height, weight, active and passive smoking, parity and folic acid supplement use were obtained from the questionnaire at enrolment in the study. FAS use was categorised into three groups: (i) started before conception, (ii) started during the first trimester and (iii) no-supplementation.^(16)^ FAS use was defined as each woman having taken 400 µg folic acid per day and greater than or equal to 5 days each week. Pregnancy outcome data included date of delivery, gestational age, neonatal gender, birth weight, birth length, head circumference, malformations, maternal height, pregnancy weight and complications of pregnancy or in delivery; details of medications used were collected after delivery by examination of the full clinical records and confirmed after review by senior clinical medical staff.

### Biological sample collection and analysis

In early pregnancy (about 6–12 weeks of gestation), blood samples (about 5 ml) of each subject were drawn via venipuncture and were collected into 6 ml, metal-free EDTA-treated tubes at the Tongliang Maternal and Children Health Care Hospital. The blood was centrifuged at 3,500 rotations per minute for 15 min at –4°C, and then the plasma and packed RBC were separated and stored at –80°C. To analyse plasma folate, RBC folate and vitamin B_12_ concentrations, plasma samples (about 1 ml) and RBC samples (about 1 ml) were selected and transported to the Department of Clinical Chemistry at the Capital Institute of Paediatrics, Beijing, China. After thawing, the plasma folate, RBC folate and vitamin B_12_ were measured with a competitive receptor binding immunoassay (Chemiluminescent Immunoenzyme Assay Access Immunoassay System; Beckman Coulter, Krefeld, Geyrman).^(17)^ The intra assay CV for serum folate and vitamin B_12_ were 3.8–6.5% and 5–7%, respectively. RBC folate was determined by a microbiological assay.^(18)^

### Birth outcomes

Information concerning gestational age at birth (weeks), offspring gender, placental weight (grams) and birthweight (grams) were obtained from hospital clinical records. The definition of SGA was a gestational age-adjusted and sex-adjusted birthweight below the tenth centile in this study cohort (<–1.79 SD),^(19)^ and prematurity was defined as a spontaneous vaginal birth of an infant before 37.0 weeks of gestation by the WHO.^(20)^

### Statistical analyses

Data were presented as the mean ± SD for the normal variables and median (P25, P75) for non-normal variables. Effects with *p*<0.05 were considered statistically significant. Two independent samples *t* tests were used to observe the differences of the plasma vitamins concentrations in both SGA and prematurity group compared with non-SGA and non-prematurity group separately. The differences in change among the three groups were estimated with ANOVA, and the percentage rates were assessed with Fisher’s probability test. Multiple linear regression models were used to estimate the effects of independent variables on head circumference, birth length and birth weight. Data were entered and analysed with the EpiData 3.0 statistical package (Centers for Disease Control and Prevention, Atlanta, GA) and the SPSS software package ver. 19.0 (SPSS Inc., Chicago, IL), respectively.

## Results

### The characteristics of the participants

We recruited 309 women, and the subjects’ characteristics are shown in Table 1. Of the total, 299 contributed a blood sample in early pregnancy (Ten were excluded for a failure in drawing blood). Among the 299 pregnant women, information on birth outcomes was retrieved after delivery only from 253 mother-infant pairs. The mean age of the women in the entire cohort was 26.8 years, and BMI was 21.1. The mean gestational age was 39.0 weeks. Of the participants, 64.4% were nulliparous, 56.9% had finished higher education and 83.2% had taken FAS, with 16.2% of the women having started FAS before conception. Among the 253 newborns, the mean birthweight was 3,310.0 g, 6.3% were preterm and 6.8% were SGA.

### Awareness and use of folic acid among pregnancy women

As is shown in Table 2, most of the pregnancy women (83.2%) received free folic acid during the pregnancy and almost all the pregnant woman (98.7%) didn’t know the role of folic acid in other congenital defect prevention. Only 16.2% pregnant women began taking folic acid 3 months before pregnancy and 1.3% knew the role of folic acid in other complications. Further, obstetricians/gynaecologists and relatives/friends were the main source of learning about folic acid.

As is shown in Table 3, there is no statistically significant association with the use of folic acid among pregnant with respect to food expenditure, employment, gravidity and multivariate-adjusted analyses revealed. With regard to the relationship between folic acid intake and selected factors, pregnant that had higher education levels, income, multiparous and age were more likely to take folic acid during preconception. In addition, pregnant who were aged 25–30 years old, high-income and multiparous were more likely to start folic acid supplementation before conception.

### Folic acid supplement intake and B vitamins levels in women

From the total study cohort, 249 (83.3%) subjects reported FAS intake and contributed a blood sample. Of that number, 49 (16.4%) started supplementation before conception, and 200 (66.9%) started supplementation during the first trimester. Maternal plasma folate and RBC folate levels were 8.60 ± 3.27 ng/ml and 618.37 ± 274.03 ng/ml in group N; 16.78 ± 5.04 ng/ml and 827.57 ± 271.92 ng/ml in group A; 13.97 ± 5.86 ng/ml and 776.14 ± 307.68 ng/ml in group B. Plasma folate and RBC folate supplementation groups were obviously higher than that of group N (*p*<0.01; Table 4). There were statistically significant differences between the three groups. The mean vitamin B_12_ levels was 903.62 ± 374.93 pg/ml in group A, significantly higher than that of 787.84 ± 302.28 pg/ml in group N and of 762.33 ± 325.60 pg/ml in group B (*p*<0.05). However, there was no significant difference between groups N and B (Table 4).

### Daily early pregnancy folic acid implementation and pregnancy outcomes

The associations between daily folic acid implementation and pregnancy outcomes are shown in Table 5. The SGA and premature incidence rates (17.5% and 15.0%) of the no-use group were higher than those in the implementation group (4.7% and 4.7%). Fisher’s probability test indicated that there were statistical differences between the two groups in both SGA and prematurity (*p*<0.05). The mean birth weight of group N was much lower than group A and B combined (3,181.3 ± 423.2 vs 3,315.4 ± 411.9). However, there was no significant difference between the two groups (*p* = 0.631). This may also suggest that folic acid plays an important role in birthweight. We speculated that it might be limited by small samples.

### Concentrations of folate and vitamin B_12_ biomarkers in cases and controls according to birth status

The associations of folate and vitamin B_12_ Concentrations in cases and controls are shown in Table 6. The mean plasma folate levels were found to be 10.08 ± 4.54 ng/ml in the SGA group and 13.64 ± 5.44 ng/ml in the control group (*p*<0.05); we also found to be 10.28 ± 5.47 ng/ml in the prematurity group and 13.59 ± 5.40 ng/ml in the control group (*p*<0.05). However, we did not found that in RBC folate and vitamin B_12_ between cases and controls.

We investigated the factors that related to physical development at birth by a multiple linear regression model in which the dependent variables were plasma folate, RBC folate and vitamin B_12_ (Table 7). The model revealed that RBC folate levels affected the infant birth weight (B = 0.197, *p*<0.01) and birth length (B = 0.149, *p*<0.05).

## Discussion

It is well-known that plasma folate, RBC folate and vitamin B_12_ have received a great deal of attention in that lower levels of them have been linked to SGA, prematurity and the risk of vascular-related pregnancy complications.^(14,21)^ Chinese government began distributing free peri-conceptional FAS (400 µg per day) to rural women, but there are few studies evaluated vitamin B_12_ and effect of the FAS program on concentrations of folate, ultimately improving and pregnancy outcomes in rural area. In the present study, we have demonstrated the positive effects on Chinese government FAS program on folic acid supplementation rate and improving pregnancy outcomes in rural area.

As is shown in Table 2, most of the pregnancy women received free folic acid during the pregnancy and knew the role of folic acid in birth defect prevention. It indicated that Chinese government FAS program had improved the pregnancy awareness of folic acid supplement, especially about the important role in birth defect prevention. However, few pregnant women began taking folic acid 3 months before pregnancy (16.2%) and know the role of folic acid in other complications during pregnancy (1.3%). Further only 11.7% of the pregnant women were learning about folic acid by media. The rates were much lower than those in developed countries.^(22–24)^ It indicated that health education should be strengthened, specially making more efforts on prepregnancy education and cooperating with public media. Agreement with other studies,^(23,25)^ the ones with higher education levels, income, multiparous and age were more likely to take folic acid before pregnancy. Therefore, preconceptional health education should take more concern on the pregnant women with lower education levels, income and nulliparous.

Our study was conducted in a rural mountain area, which had distributed free periconceptional folic acid supplements (400 µg per day); this enabled us to accurately assess the consumption of folate supplements and the association with subsequent adverse pregnancy outcomes, including SGA and prematurity. In contrast, previous studies were conducted in the general population to find out the association between vitamin B levels with some adverse pregnancy outcomes.^(21,26)^ The consumption of folate supplements among study subjects and the correlations with maternal vitamin B levels could not be completely ruled out.^(27–29)^

In this cohort study of pregnant women who resided in Tongliang, Chongqing, 83.2% reported taking folic acid supplementation during the periconceptional period; 67.0% started folic acid use during the first trimester, and 16.2% of the women began taking it before conception. Recently, another survey conducted in China also revealed that more than 80% of women take folic acid supplementation during the periconceptional period.^(25)^ The rates are similar with develop countries, such as Canada (91.3%),^(23)^ the USA (73.9%)^(24)^ and the Netherlands (86%).^(22)^ This indicate that the FAS program in China had greatly help to periconceptional folic acid supplementation and the supplemental rate among the Chinese population increased over time. Meanwhile, our findings confirmed that the women taking folic acid supplementation during the periconceptional period tended to have timely initiation of prenatal care, higher incomes, higher education, and planned pregnancies.

As is shown in Table 4–6 that the lower plasma folate, RBC folate and vitamin B_12_ concentrations in early pregnancy in the no-supplementation group were associated with lower birth weight and a higher risk of having an SGA infant and of prematurity. Furthermore, women who delivered SGA or premature infants had reduced plasma folate compared with the control group. To our knowledge, this is one of the few prospective studies that conducted among women planning to conceive that has shown the correlations between the practice of folic acid supplementation and the maternal blood folate concentrations in early pregnancy. Moreover, we did establish a significant association between folic acid supplementation and lower risk of having an SGA infant or premature birth (Table 5), It is consistent with previous studies.^(16,30,31)^

Vitamin B_12_ acts as a cofactor for enzyme reactions in one-carbon metabolism and plays a vital role in folate circulation. This is not surprising given the critical role vitamin B_12_ plays in folate metabolism. Data on the impact of maternal blood concentration of vitamin B_12_ are conflicting. As Sutton *et al.*^(32)^ reported, vitamin B_12_ levels were not lower in women whose conceptuses had NTDs or other types of defects. However, Molloy *et al.*^(33)^ have also found NTD pregnancies to be associated with low maternal levels of vitamin B_12_ independent of folate status. In the present study, we did not found any association between maternal vitamin B_12_ concentration in early pregnancy and SGA, as well as prematurity (Table 6). This might be explained by the fact that folate is a substrate and vitamin B_12_ serves as a cofactor in the homocysteine metabolism, and so is not often a limiting factor.^(34)^ Furthermore, it is also possible that diet-gene interactions are involved and small number of pregnancy outcomes. We hope that obtaining dietary and genotype data for other Chinese populations will shed light on this unexpected correlation.

In a multiple linear regression model (Table 7), we found that lower concentrations of folate may result in a relative lighter tendency of birth weight and shorter tendency birth length. However, we had not observed the differences of the levels of RBC folate between cases and controls of SGA or prematurity (Table 6). But it’s noteworthy “a relative lighter birth weight” is not equal to “small for gestational age at birth (SGA)”. So although there was not observed the differences of the levels of RBC folate on the SGA between the cases and controls, we still consider it’s a positive result. Previous research had shown high concentrations of maternal folate levels have an effect on birth weight but not on birth length.^(26)^ Another study found that iron-folic acid supplements were significantly associated with increased birth length compared with folic acid alone and that multiple vitamins administered together result in a significant improvement in physical development.^(35)^ Therefore, our results may be explained by RBC folate concentrations changing slowly; it provides a better indication of long-term folate status, as women with high RBC folate concentrations have a better nutritional status.

Nonetheless, there are some limitations to our study. The study’s subjects were not randomly selected: those women who came to seek early prenatal care may be more conscientious about their health and that of their unborn babies, or they may enjoy a higher socioeconomic status. This would result in an overestimation of folic acid use and blood folate levels. Furthermore, although the subjects had typical lifestyles representative of the farmers in Chongqing municipality directly under the central government, it may be difficult to extrapolate the conclusion to other rural areas in China. The most serious limitation of this study is the small number of pregnancy outcomes. In addition, our analyses were restricted by the lack of reliable dietary data and measures of maternal nutritional and health status, including maternal weight gain and blood pressure, later in pregnancy.

In summary, we found significant associations between FAS in rural women of childbearing age and maternal folate and vitamin B_12_ levels, the higher folate and vitamin B_12_ concentration and lower risk of SGA infant and prematurity in early pregnancy in women with FAS intake compared with no-use women in early pregnancy. Furthermore, the mean of maternal plasma folate concentrations in early pregnancy in women who delivered SGA infant or premature infants were lower compared to the control group. In addition, health education should be strengthened, specially making more efforts on prepregnancy education and cooperating with public media.

## Figures and Tables

**Table 1:** Demographics and clinical characteristics of the subjects

Characteristic	Value *n* (%)
**Pregnancy characteristics (***n*** = 309)**	
Maternal age (years)	
<25	134 (43.4)
25–30	118 (38.2)
>30	57 (18.4)
BMI index before pregnancy (kg/m^2^)	
<18.5	58 (18.8)
18.5–24.9	218 (70.6)
≥25	33 (10.6)
Monthly income of the family (RMB)	
<3,500	75 (24.3)
3,500–4,500	177 (57.3)
>4,500	57 (18.4)
Gravidity	
Primigravida	101 (32.7)
Multigravida	208 (67.3)
Parity	
Nulliparous	199 (64.4)
Multiparous	110 (35.6)
Education	
Primary school or lower	15 (4.9)
Secondary school	214 (69.3)
University or above	80 (25.8)
Employment	
No	217 (70.2)
Yes	92 (29.8)
Passive smoking exposure	
No	108 (35.0)
Yes	201 (65.0)
**Neonate characteristics (***n*** = 253)**	
Birth length	50.3 ± 1.4*
Head circumference	33.9 ± 0.9*
Birth weight (g)	3,310.0 ± 413.0*
LBW	7 (2.8)
SGA	
<10th percentile	17 (6.8)
LGA	
>90th percentile	15 (6.0)
Preterm	
<37 weeks	16 (6.3)

*Data were shown as the mean ± SD.

**Table 2:** Awareness and use of folic acid among pregnancy women in the cohort study

Items	Value *n* (%)
Enrolled subjects	309
Received free folic acid	
No	52 (16.8)
Yes	257 (83.2)
Know about the role of folic acid in birth defect prevention	
No	68 (22.0)
Yes	241 (78.0)
Know about the role of folic acid in other complications during pregnancy	
No	305 (98.7)
Yes	4 (1.3)
Means of learning about folic acid	
Media	36 (11.7)
Obstetricians and gynaecologists	158 (51.1)
Relatives and friends	98 (31.7)
Others	17 (5.5)
Previous thought about correct time of folic acid supplement	
Before pregnancy	171 (55.3)
Early in pregnancy	33 (10.7)
In late pregnancy	3 (1.0)
Do not know	102 (33.0)
Began taking folic acid	
Never	52 (16.8)
Early in pregnancy	207 (67.0)
3 months before pregnancy	50 (16.2)
Times of folic acid supplement each week	
1–3	4 (1.3)
3–4	8 (2.6)
≥5	257 (83.2)

**Table 3:** Descriptive, univariate, and multivariate analyses of all enrolled pregnant concerning the use of folic acid

	Supplement of folic acid			Took folic acid during the first trimestery			Took folic acid before pregnancy		
	*n* (%)	Unadjusted OR^a^ (95% CI)	Adjusted OR^a^ (95% CI)	*n* (%)	Unadjusted OR^a^ (95% CI)	Adjusted OR^a^ (95% CI)	*n* (%)	Unadjusted OR^a^ (95% CI)	Adjusted OR^a^ (95% CI)
All enrolled pregancy women	257 (83.2)			207 (70.0)			50 (16.2)		
Age (years)									
<25	105 (40.8)	1	1	92 (44.4)	1	1	13 (26.0)	1	1
25–30	101 (39.3)	1.7 (0.78–3.71)	1.66 (0.58–4.69)	77 (37.2)	2.18 (1.16–4.14)*	7.83 (2.11–28.99)*	24 (48.0)	1.48 (1.21–4.10)*	5.54 (1.71–17.94)*
>30	51 (19.9)	1.32 (0.62–2.81)	1.07 (0.46–2.52)	38 (18.4)	1.82 (1.66–2.25)*	4.113 (1.31–12.91)*	13 (26.0)	0.98 (0.46–2.08)	1.74 (0.77–3.92)
Education									
primary shool or lower	12 (4.7)	1	1	10 (4.8)	1	1	2 (4.0)	1	1
Secondary school	171 (66.5)	3.09 (0.68–14.08)	0.28 (0.05–1.52)	140 (67.6)	0.81 (0.25–2.62)	1.13 (0.17–7.29)	31 (62.0)	0.57 (0.12–2.78)	1.287 (0.22–7.69)
University or above	74 (28.8)	1.7 (1.27–7.58)*	4.25 (1.51–12.99*	57 (27.6)	0.76 (0.44–1.34)	0.87 (0.37–2.07)	17 (34)	0.63 (0.33–1.21)	1.07 (0.21–5.49)
Employment									
No	180 (70.0)	1	1	150 (72.5)	1	1	30 (60.0)	1	1
Yes	77 (30.0)	0.95 (0.49–1.83)	1.24 (0.60–2.60)	57 (27.5)	1.38 (0.83–2.29)	1.27 (0.55–2.63)	20 (40.0)	0.58 (0.31–1.08)	1.2 (0.57–2.54)
Monthly income (RMB)									
<3,500	62 (24.1)	1	1	53 (25.6)	1	1	9 (18.0)	1	1
3,500–4,500	144 (56.0)	1.67 (0.20–13.98)	4.1 (0.35–47.81)	117 (56.5)	3.89 (0.70–21.75)	4.97 (0.63–39.22)	2,754.0)	0.17 (0.02–1.87)	0.429 (0.03–5.83)
>4,500	51 (19.9)	1.08 (0.23–5.15)	2.59 (0.44–15.04)	37 (17.9)	1.658 (0.49–5.57)	2.05 (0.39–10.74)	14 (28.0)	1.52 (1.13–5.04)*	1.814 (1.17–3.863)*
Food expenditure (RMB)**									
<1,500	34 (13.2)	1	1	26 (12.6)	1	1	8 (16.0)	1	1
1,500–2,000	195 (75.9)	0.43 (0.03–5.53)	0.12 (0.00–3.68)	157 (75.8)	0.833 (0.07–10.27)	0.4 (0.09–1.74)	38 (76.0)	0.4 (0.78–3.71)	2.5 (0.60–10.44)
>2,000	28 (10.9)	1.08 (0.42–2.76)	1.04 (0.38–2.82)	24 (11.6)	0.829 (0.38–1.81)	0.8 (0.24–2.66)	4 (8.0)	1.53 (0.51–4.54)	1.392 (0.43–4.49)
Gravidity [*n*(%)]									
Primigravida	170 (66.1)	1	1	134 (64.7)	1	1	36 (72.0)	1	1
Multigravida	87 (33.9)	1.55 (0.85–2.84)	1.3 (0.52–3.26)	73 (35.3)	1.05 (0.6–14.71)	1.66 (0.73–3.76)	14 (28.0)	1.51 (0.78–2.95)	3.96 (1.48–10.60)
Parity [*n*(%)]									
Nulliparous	84 (32.7)	1	1	72 (34.8)	1	1	12 (24.0)	1	1
Multiparous	173 (67.3)	1.08 (0.53–1.89)	1.42 (0.56–3.57)	135 (65.2)	1.34 (1.20–2.25)*	4.69 (1.51–14.51)*	38 (76.0)	1.6 (1.30–2.21)*	4.08 (1.11–11.57)*

OR, odds ratio; CI, confidence interval; 1, reference layer. ^a^Adjusted ORs were derived from multivariate logistic regression; unadjusted ORs were derived from univariate logistic regression. *p<0.05. **Monthly food expenditure of the family (RMB).

**Table 4:** Concentrations of folate and vitamin B_12_ biomarkers in those who did and did not supplement with folic acid (*n* = 299)^a^

Biomarker	Daily folic acid implementation			*p* value
	No-use (group N)	Start before conception (group A)	During the first trimester (group B)	
N	50	49	200	
Plasma folate (ng/ml)	8.60 ± 3.27	16.78 ± 5.04^c^	13.97 ± 5.86^d^^,^^e^	0.000
RBC folate (ng/ml)	618.37 ± 274.03	827.57 ± 271.92^c^	776.14 ± 307.68^d^	0.001
Vitamin B_12_ (pg/ml)	787.84 ± 302.28	903.62 ± 374.93	762.33 ± 325.60^e^	0.028

^a^All values are mean ± SD. *N* represents the number of subjects with data available. ^b^The differences in change between three groups were estimated with ANOVA. ^c^*p*<0.01 when group A compared with group N. ^d^*p*<0.01 when group B compared with group N. ^e^*p*<0.01 when group A compared with group B.

**Table 5:** Associations between daily folic acid implementation and pregnancy outcomes

	Daily folic acid implementation		*p* value^a^
	No-use (*n* = 40)	folic acid implementation (*n* = 213)	
SGA^b^	7 (17.5)	10 (4.7)	0.009
Prematurity^b^	6 (15.0)	10 (4.7)	0.025
Birth weight^c^ (g)	3,181.3 ± 423.2	3,315.4 ± 411.9	0.631

^a^The percentage rates were assessed with fisher probabilities test or independent samples *t* test between the two groups. ^b^Values were *n* (%). ^c^Data were shown mean ± SD.

**Table 6:** Concentrations of folate and vitamin B_12_ biomarkers in cases and controls according to birth status^a^

Biomarker	SGA birth		*p*^b^	Prematurity		*p*^c^
	Cases (*n* = 16)	Controls (*n* = 229)		Cases (*n* = 15)	Controls (*n* = 231)	
Plasma folate (ng/ml)	10.08 ± 4.54	13.64 ± 5.44	0.011	10.28 ± 5.47	13.59 ± 5.40	0.022
RBC folate (ng/ml)	765.58 ± 270.51	768.02 ± 311.92	0.976	740.97 ± 311.78	767.36 ± 307.45	0.749
Vitamin B_12_ (pg/ml)	808.67 ± 306.38	775.72 ± 341.17	0.707	732.50 ± 311.44	783.78 ± 339.79	0.570

^a^All values are mean ± SD. *N* represents the number of subjects with data available. SGA defined as, 10th customised birth weight percentile. Prematurity defined as a live birth with a gestational age <37 weeks gestation. The numbers of case and control subjects vary slightly because of missing values for some variables. ^b^*p* values between SGA and non-SGA births, by independent samples *t* test between the two groups. ^c^*p* values between prematurity and non-prematurity births, by independent samples *t* test between the two groups.

**Table 7:** Associations between early pregnancy biomarker concentrations and head circumference, birth length and birth weight^a^

Blood nutrients	Head circumference		Birth length		Birthweight	
	95% CI^b^	*p* value	95% CI^b^	*p* value	95% CI^b^	*p* value
Serum folate (ng/ml)	0.060 (–0.012, 0.031)	0.373	–0.032 (–0.040, 0.024)	0.625	–0.090 (–16.773, 3.192)	0.178
RBC folate (ng/ml)	–0.016 (0.000, 0.000)	0.82	0.197 (0.000, 0.001)	**0.003**	0.149 (0.017, 0.381)	**0.032**
Vitamin B_12_ (pg/ml)	0.464 (0.000, 0.000)	0.464	–0.086 (–0.001, 0.000)	0.169	–0.041 (–0.205, 0.104)	0.523

^a^Multivariate linear regression analysis with head circumference, birth length and birthweight as dependent variables and Serum folate, RBC folate and vitamin B_12_ concentrations as independent variables. ^b^All values in this column are regression coefficients (95% CI) and their corresponding B value.
